# A warning system for urolithiasis via retrograde intrarenal surgery using machine learning: an experimental study

**DOI:** 10.1186/s12894-022-01032-5

**Published:** 2022-06-06

**Authors:** Jinho Jeong, Kidon Chang, Jisuk Lee, Jongeun Choi

**Affiliations:** 1 School of Mechanical Engineering, Yonsei University, Seoul, Republic of Korea; 2 Department of Urology, Wonju College of Medicine, Yonsei University, Wonju, Republic of Korea; 3 MODULABS, Seoul, Republic of Korea

**Keywords:** Urolithiasis, Kidney stones, Retrograde intrarenal surgery, Discrete wavelet transform, Machine learning

## Abstract

**Background:**

To develop a warning system that can prevent or minimize laser exposure resulting in kidney and ureter damage during retrograde intrarenal surgery (RIRS) for urolithiasis. Our study builds on the hypothesis that shock waves of different degrees are delivered to the hand of the surgeon depending on whether the laser hits the stone or tissue.

**Methods:**

A surgical environment was simulated for RIRS by filling the body of a raw whole chicken with water and stones from the human body. We developed an acceleration measurement system that recorded the power signal data for a number of hours, yielding distinguishable characteristics among three different states (idle state, stones, and tissue–laser interface) by conducting fast Fourier transform (FFT) analysis. A discrete wavelet transform (DWT) was used for feature extraction, and a random forest classification algorithm was applied to classify the current state of the laser-tissue interface.

**Results:**

The result of the FFT showed that the magnitude spectrum is different within the frequency range of < 2500 Hz, indicating that the different states are distinguishable. Each recorded signal was cut in only 0.5-s increments and transformed using the DWT. The transformed data were entered into a random forest classifier to train the model. The test result was only measured with the dataset that was isolated from the training dataset. The maximum average test accuracy was > 95%. The procedure was repeated with random signal dummy data, resulting in an average accuracy of 33.33% and proving that the proposed method caused no bias.

**Conclusions:**

Our monitoring system receives the shockwave signals generated from the RIRS urolithiasis treatment procedure and generates the laser irradiance status by rapidly recognizing (in 0.5 s) the current laser exposure state with high accuracy (95%). We postulate that this can significantly minimize surgeon error during RIRS.

## Introduction

For many years, endourologists have been searching for a more efficient, less traumatic treatment for urolithiasis. As results, novel and innovative instruments have been developed, expanding the treatment armamentarium. RIRS has gained acceptance as the first treatment alternative for renal stones sized up to 20 mm and in other specified circumstances [1]. RIRS studies investigated the surgical outcomes for overall population have verified the impact of certain surgical and medical complications [2] or renal injury [3]. Although RIRS is widely accepted as minimally invasive treatment, its complications and possible renal damage must be dealt with. The aim of this study was to develop a technology of machine learning-based early warning system to prevent kidney and ureter damage during RIRS for urolithiasis to minimize surgical error and hence improve patient outcomes. To the best of our knowledge, this is the first study to apply discrete wavelet transform (DWT) data feature engineering and machine learning (ML) techniques from RIRS-generated data to minimize the surgical error. Recently, artificial intelligence (or ML) have resulted in a paradigm shift for clinical decision support systems. Clinicians and surgeons can now make precise diagnosis supported by reliable and accurate ML models, resulting in anticipated and improved postoperative outcomes. Several studies conducted on ML applications in urology over the past years have addressed possible improved patient outcomes in the various urologic area, such as renal cell carcinoma [4], patient-specific urologic surgical care [5], prostate cancer [6], etc. However, existing studies have not considered what we believe to be the crucial part of treatment: the procedure itself. One has differentiated distal ureteral stones and pelvic phleboliths convolutional neural network on CT scans [7]. In terms of urolithiasis treatment, the postoperative outcomes of percutaneous nephrolithotomy were predicted using Fisher discriminant analysis [8] and support vector machine [9]. Similarly, the stone-free status after shockwave lithotripsy has been obtained using clinical information and CT image with an artificial neural network [10] and a decision tree method [11, 12]. There also has been an attempt to predict the stone-free rate (SFR) prior to RIRS with the R.I.R.S scoring system and a statistical approach [13]. In terms of statistical approach, [14] investigated the ureteroscopy plus elective double-J stent treatment using a multivariate analysis of factors predict hospitalization. The referenced study investigators assumed that all the procedures were routinely conducted and would only predict outcomes from the procedures. We believe that the optimal method to enhance prognosis after a procedure is to minimize errors during that procedure. To achieve improved outcomes, we propose a novel early warning system for RIRS detecting unexpected laser-tissue interface. The system measures the shockwave generated from the stone or tissue contacted with laser for brief time intervals. Based on the short period of the recorded signal, the early warning system classifies the time-series signal and advises the surgeon of the current state with greater than 95% accuracy with proper settings. With this proposed method, the surgeon can promptly respond to the laser irradiance status of our warning system, minimizing the chances of ureter damage.

## Methods

### Data collection

Data recording of patient treatments was discouraged due to the potential of it affecting the procedure. Therefore, we simulated RIRS as much as possible to collect data accordingly. After filling the body of a raw whole chicken body with water, stones from humans were placed inside the chicken body and crushed with the laser.

**Fig. 1:**
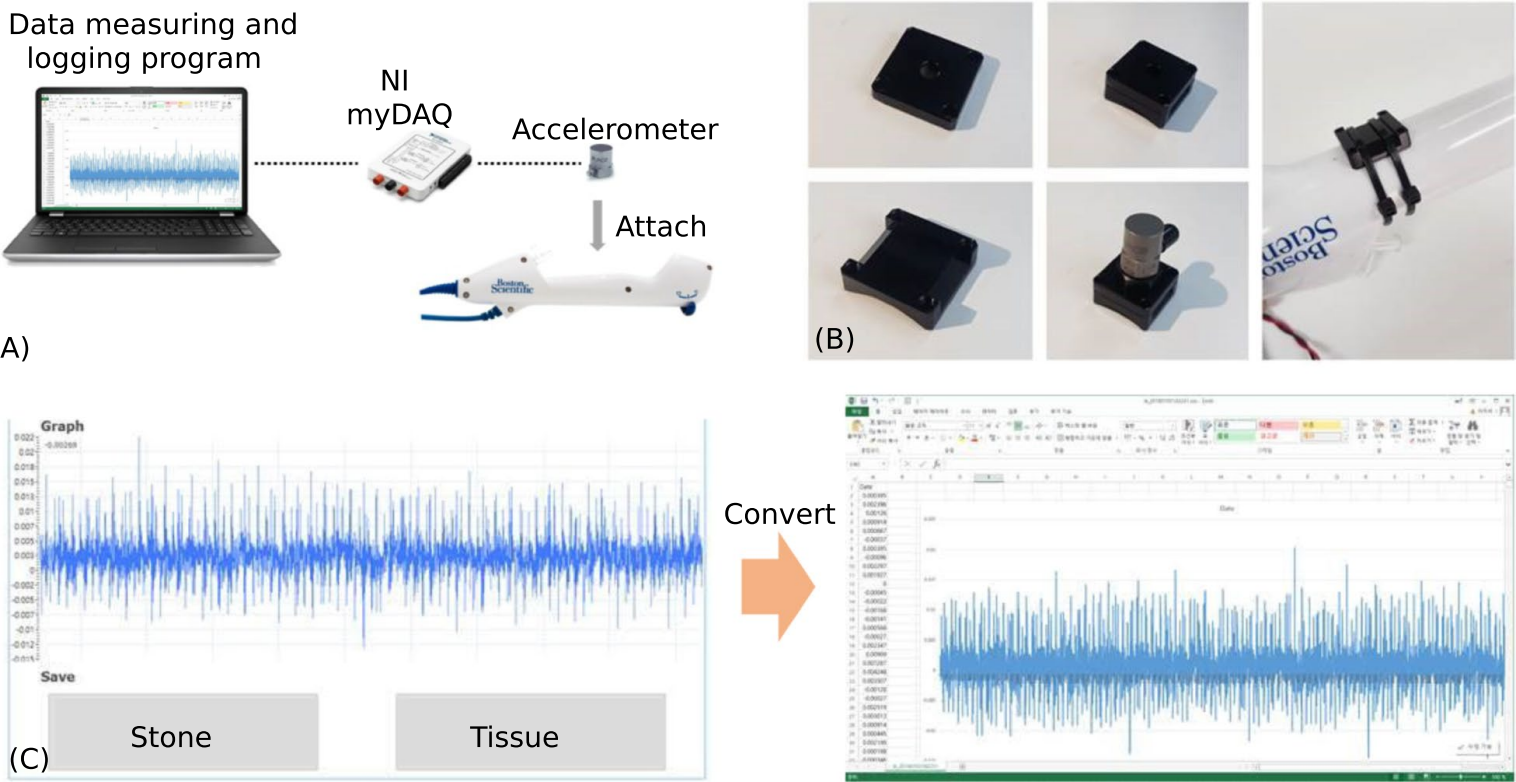
Depiction of the data collection system.** a** Illustration of the overall acceleration measure system. **b** Accelerometer and its mount attached to an endoscope. **c** Data storage and conversion with the data measuring program

Our hypothesis was that different shockwaves are generated depending on the laser interface with the stone or tissue. Based on this hypothesis, an acceleration measurement system was developed and attached near the endoscope handle. A detailed depiction of data collection system is shown on Fig. 1. An accelerometer mount placed between the endoscope and accelerometer was carefully designed via 3D printer to prevent instable data logging (Fig. 1a, b). Through the accelerometer, the magnitude of the force transmitted to the hand of a surgeon can be measured at a rate of 100 samples per second (samples every 10 ms). During the simulated RIRS, the surgeon will indicate when the laser interfaces with the tissue or stone by pressing the button on the data measuring program handle so the data can be logged. The logged data is then converted into the Microsoft Excel (.xlsx) format (Fig. 1c).

The data collection was conducted for approximately 18 hours total, which contains the wave signals from the idle state, laser-stones, and laser-tissue interface. These signals generate single, long time-series data. Therefore, before we applied our method to the collected data, we splitted the data to have a certain timestep long. The length of the timestep was set to 50 steps (i.e., 500 ms) throughout this paper. This hyperparameter value was obtained by a trial-and-error process while considering the trade-off relationship between the accuracy performance and shorter timestep. A shorter timestep is preferred because it promptly provides feedback to the surgeon to be reflected in the procedure in real time.

### Hypothesis validation

The irradiance of laser rays leads to stone heating and stone water vaporization, forming a vapor bubble around the stone. This vapor bubble expands and collapses rapidly and induces pressure transients followed by shockwaves, culminating in stone fragmentation [15]. The shockwave can also be generated during the soft-tissue laser ablation, but we expect that the generated waveform should be different from that at the laser-stone interface [16].

The fast Fourier transform (FFT) is an algorithm that calculates the (discrete) Fourier transform of the time-serial signal, and it was conducted on the collected dataset. The FFT converts a time domain signal into a representation in the frequency domain, indicating that we can determine the frequencies that the signal consists of as well as the dominant frequencies.

**Fig. 2:**
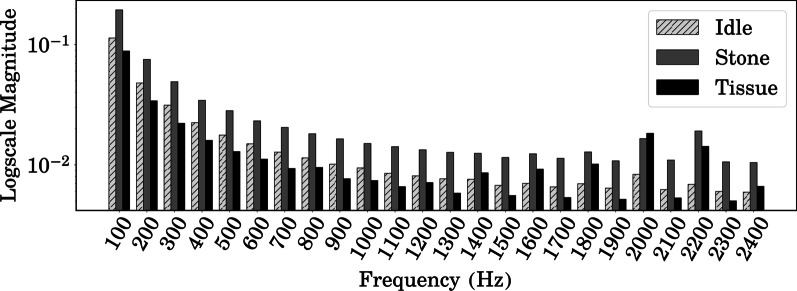
Fast Fourier transform results for the collected dataset

In every three cases, not shooting the laser, stone, and tissue are hit by the laser, are named as “Idle,” “Stone,” and “Tissue,” respectively. All of the data were sliced into segments of 500 ms (50 timesteps) that were determined by the performance assessment. Every segment was analyzed by FFT and averaged within each case (Fig. 2 shows the result of the FFT analysis with a log-scale vertical axis). The result indicates that there are distinct differences among the three states. Representatively, The case of “Stone” showed the largest magnitude in most of the frequency range. In other words, the signals from different laser irradiance states can be distinguished based on their waveform. Although we can differentiate the signal data, FFT merely shows this information. The difference between stationary and non-stationary signals cannot be determined from the FFT result, which have time-invariant and variant frequency components, respectively. RIRS for urolithiasis naturally involves a non-stationary (frequency component: variant) signal-generating circumstance. This was the rationale for this study considering DWT as a feature engineering method, which will be described in detail.

### Overall process of the algorithm

In this study, the ML model process can be divided into two parts: feature engineering with DWT and classification with the random forest model. DWT enables the classifier to collect more information from time-series waveform data. The random forest classifier was accepted as a classifier model throughout this study. All the algorithms are implemented on a machine with Intel^®^i9-9900K and Nvidia^®^RTX2080ti and written in Python 3.6.3.

#### DWT

DWT is a type of wavelet transform for wavelets that are discretely sampled, such as our collected data. The major advantage of DWT is its ability to capture both frequency and time zone information by converting the signal into a *family* of wavelets [17].

The wavelet family defined by a *mother* wavelet function $\Psi$. There also are *child* wavelets determined by $\Psi$ and its parameter *j* and *k*. The formal definition of discrete set of child wavelet is as follows:

$$\begin{aligned} \Psi _{j,k}= & {} 2^{-\frac{j}{2}}\Psi (2^{j}t-k)\\ \gamma _{j,k}= & {} \int _{-\infty }^{\infty } x(t) \Psi _{j,k} dt \end{aligned}$$

This $\gamma _{j,k}$ is a convolution of *x*(*t*) with a dilated, reflected, and normalized variant of $\Psi$ if we see $\gamma$ as a function of *k* only.

**Fig. 3:**
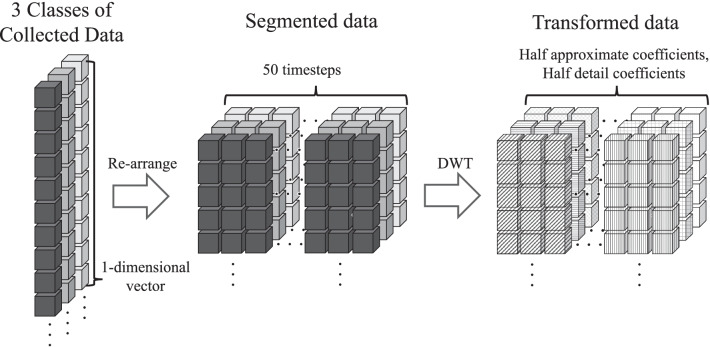
An illustration of the data transformation process

We utilized a single level (order) of the transform which outputs approximate and detail coefficients, each having a length of half the original data length if no padding was applied to the data segments. These coefficients become our transformed data that is fed into the random forest machine learning model. Fig. 3 illustrates the whole data transformation as a feature engineering process.

DWT has been widely used in numerous studies using time-series data, which include signal processing, classification, and detection. Among those applications, DWT is recognized for its effectiveness in electroencephalogram (EEG), electrocardiogram, and electromyographic (EMG) signal analysis. DWT was utilized for extracting features from possibly contaminated EMG signals [18] and as a feature extraction method for emotion recognition from EEG signals [19].

DWT is the process of decomposing a given signal into *wavelet bases.* The decomposition into wavelet bases is referred to as *multiresolution analysis* [20]. We used a single level of the transform that generates approximate and detail coefficients, each having a length of half the original data length if no padding was applied to the data segments. Further, we used various types of discrete wavelet family, such as Haar, Daubechies, reverse biorthogonal, and discrete Meyer, referred to as *haar*, *db*, *rbio*, and *dmey*, respectively. The number followed by the wavelet is the distinction of the approximation orders of that family. Each data segment with a length of 50 timesteps (500 ms) is transformed through DWT and converted to approximate and detail coefficients. The coefficients are used as a dataset to train and test the random forest ML classifier.

#### Random forest classifier

Random forest, also known as random decision forest, is an ensemble-learning [21] method for classification and many other tasks that composed with a multitude of decision trees at a training stage and output the class that is the label of the classes when it comes to a classification task testing stage [22, 23]. A random forest is a meta estimator that trains several decision tree classifiers on sub-sample sets and averages the result to increase predictive accuracy and reduce the chance of over-fitting. In this study, we implemented the classifier using the scikit-learn [24] Python library with its default hyperparameters.

To validate the model performance correctly, *m* segments were picked from each class, with slight discrepancy in data amount among them. With the m segments, *k*-fold cross-validation process was conducted, which separates the training and test data for independent use for each process. These processes were repeated for *n* times and the outputs of the random forest classifier performance metric, accuracies, were averaged to evaluate the general performance. Hence, the performance can be assessed for $m\cdot n\cdot k$ different random data subset combinations. In this study, we set *m*, *n*, and *k* to 20, 100, and 10, respectively, which is basically 10-fold stratified cross-validation [25]. In short, we trained and tested the model with 20,000 different data subset combinations, averaged the accuracy outputs, and used it as a representative value for a single run.

## Results

Performance assessment will be presented in two parts: predictive accuracies and receiver operating characteristic (ROC) curves.

### Average prediction accuracies from cross-validation

With the discrete wavelets and cross-validation schemes, we obtained an average accuracy performance for each selected wavelet (Table 1).

**Table 1:** Accuracy performance metrics (averaged accuracies and their standard deviations) for each selected wavelet

Predictive accuracy	Harr	db2	db4	rbio2.4	dmey
Mean	0.939	0.948	0.946	0.945	**0.950**
Std. Dev.	0.016	0.014	0.015	0.015	0.015

A case with a dmey wavelet shows the best performance in terms of the accuracy metric (marked in bold)

**Fig. 4:**
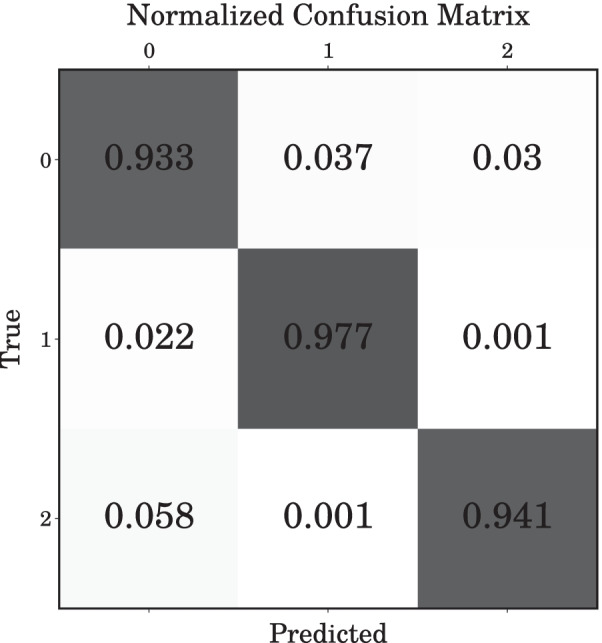
Normalized confusion matrix for the 3-class classification problem

There were no significant differences between the wavelets, but discrete Meyer achieved the best performance with a 95% prediction accuracy. This implies that the surgeon can receive information about the current laser exposure status within 0.5 s after the start of the procedure with 95% accuracy. Fig. 4 shows the results as a form of normalized confusion matrix (0, 1, and 2 denotes “Idle”, “Stone”, and “Tissue” states, respectively).

### ROC metric evaluation including dummy data procedure

ROC curve evaluation was conducted with discrete Meyer wavelet, achieving the best accuracy performance. The process for the predictive accuracy evaluation was repeated for ROC curves, as they were computed at every step and averaged. Each plot includes 1 standard deviation range accordingly. In contrast to the accuracy assessment, ROC calculation was conducted with three one-versus-rest cases as follows: Idle vs. Stone + Tissue, Stone vs. Idle + Tissue, and Tissue vs. Idle + Stone.

**Fig. 5:**
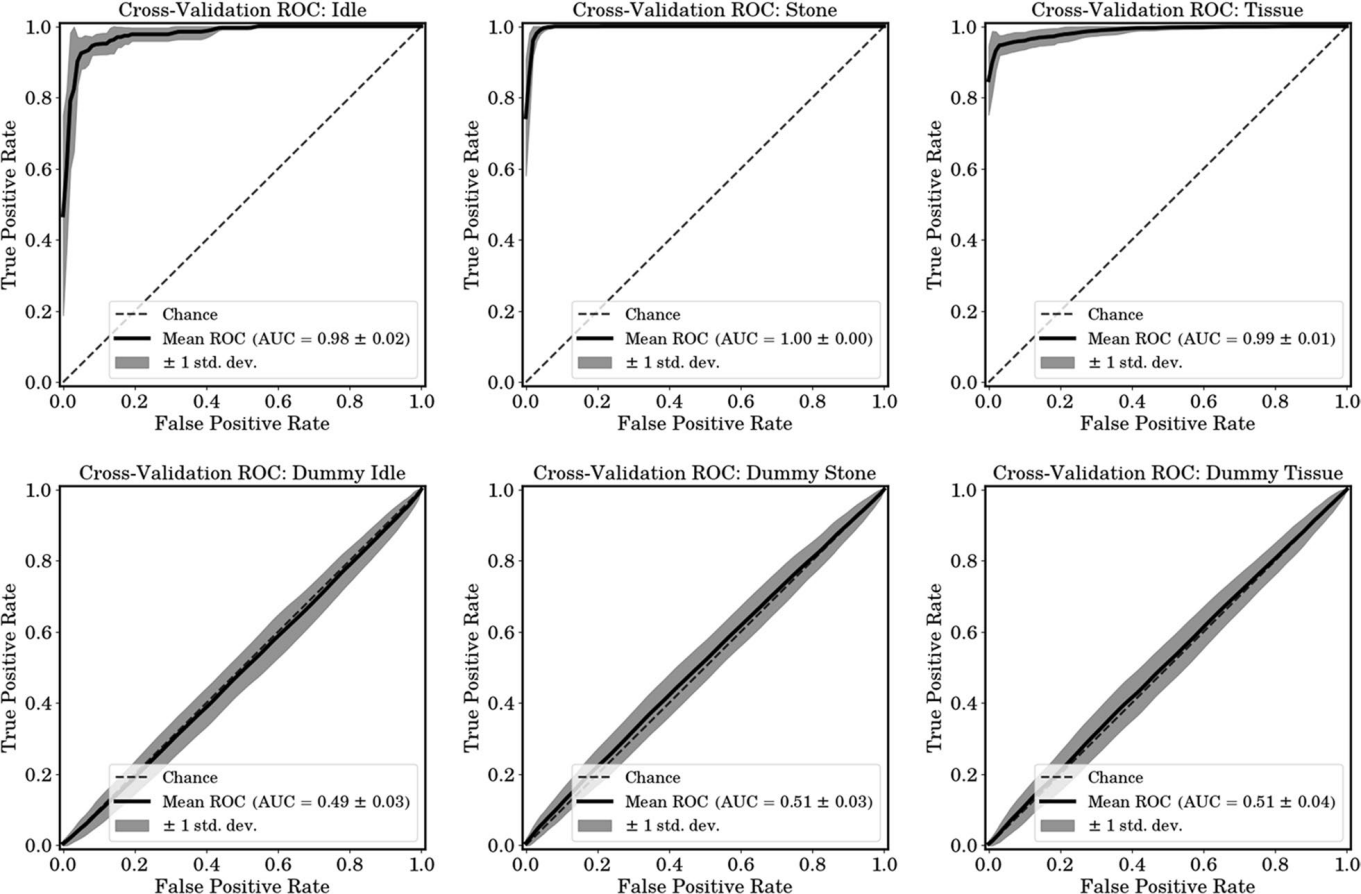
Receiver operating characteristic curves for each case

In a multi-class classification problem, the dataset is naturally imbalanced if the problem is handled as a one-versus-rest case. Hence, we generated uniformly distributed random dummy data for each class label and conducted exact same process as in the original data case. Fig. 5 shows both original and dummy data ROC curves.

All ROC curves with the original data show AUC scores of greater than 0.98 and dummy data ROC curves show scores of approximately 0.5, i.e., the data are indistinguishable and there is no bias caused in the proposed method.

The time required for the trained model to classify the incoming data is negligible (less than 10 ms on our machine). This indicates that the surgeon can receive reliable feedback in approximately 0.5 a when unwanted laser irradiation occurs during the procedure.

## Discussion

Urolithiasis is an increasingly prevalent condition worldwide for which RIRS intervention use has also increased, but there are minimal studies on its safety [2, 26]. The main RIRS complications include fever, flank pain, urinary tract infection, transient hematuria, acute urinary retention, ureteral and pelvicalyceal abrasion, stone street, subcapsular hematoma, forniceal rupture, extravasation, urinoma, ureter avulsion, bleeding requiring transfusion, and sepsis [2, 27]. Reported complication rates vary between 0 and 25% in previous studies [28].

Most complications are prevented by placing a ureteral stent after surgery. However, urinary tract injuries and perforations occur during surgery, and these injuries can cause bleeding and tissue damage, even if the complications develop gradually. In terms of that, we developed a monitoring system to reduce possible negative postoperative outcomes. Nevertheless, there are some areas of improvement as follows:

- *Acquiring Quality Data* Our warning system is currently developed with data acquired from RIRS simulation. This will be improved and developed based on data obtained through human experiments in the future.
- *Shorter Processing Time* If the processing time can be shortened even further from 500 ms, this will lead to enhanced error correction, resulting in improved prognosis.
- *Feedback Modality* There are three widely accepted feedback modalities: Visual, Auditory, and Tactile. These modalities can be utilized to provide feedback to surgeons using the warning system. An interesting future investigation would be to determine which modality is most effective in providing warning to the surgeon.
- *Dataset from Real Case* A limitation of the study is that animal experiments were conducted, and the results can be analyzed by extrapolating the data and applying it to actual surgery in the future.
- *Further applications* Presented approach can be applied to other treatments that are exploiting laser vaporisation, such as Benign Prostatic Hyperplasia (BPH) surgery [29] and MOSES technology LEP (MoLEP) for benign prostate enlargement (BPE) treatment [30].

Despite of these possible improvements that are yet to be realized, we believe our monitoring system that inputs the shockwave signal generated from RIRS for urolithiasis and reports the laser irradiance status, which rapidly recognizes (approximately 0.5 s) the current laser exposure status with high accuracy (greater than 95%), can aid both the surgeons and patients greatly.

## Data Availability

The dataset (and FFT analysis Python script) generated and analysed during the current study is available in the GitHub repository (https://github.com/MLCS-Yonsei/RIRS).
